# Supplementary material for: A Bayesian framework to unravel food, groundwater, and climate linkages: A case study from Louisiana
Source: PLoS One. 2020 Jul 30;15(7):e0236757. doi: 10.1371/journal.pone.0236757 (PMC7392305; doi:10.1371/journal.pone.0236757)
Supplement: S1 Table — (DOCX) [file pone.0236757.s001.docx]

**S1 Table.** **Correlation* matrix for the explanatory variables**

| (Pearson r / *P) | AP | GW | Iwells | PDSI | Seasonal Rainfall | Annual Rainfall | Tmean | OilPrice |
| --- | --- | --- | --- | --- | --- | --- | --- | --- |
| Area Planted | 1 | 0.45 | 0.12 | 0.01 | 0.05 | 0.09 | 0.28 | -0.01 |
| GW level | 0.00 | 1 | -0.02 | -0.03 | 0.01 | 0.05 | 0.20 | 0.02 |
| Irrigation wells | 0.01 | 0.67 | 1 | -0.10 | -0.09 | -0.13 | -0.14 | 0.07 |
| PDSI | 0.83 | 0.45 | 0.04 | 1 | 0.65 | 0.57 | -0.18 | 0.02 |
| Seasonal Rainfall | 0.30 | 0.80 | 0.07 | 0.00 | 1 | 0.68 | -0.08 | 0.03 |
| Annual Rainfall | 0.04 | 0.24 | 0.00 | 0.00 | 0.00 | 1 | 0.09 | -0.07 |
| Temp mean | 0.00 | 0.00 | 0.00 | 0.00 | 0.08 | 0.04 | 1 | 0.07 |
| Oil Price | 0.74 | 0.72 | 0.14 | 0.66 | 0.45 | 0.09 | 0.11 | 1 |

* shaded area represents P values
