# Supplementary material for: A Bayesian framework to unravel food, groundwater, and climate linkages: A case study from Louisiana
Source: PLoS One. 2020 Jul 30;15(7):e0236757. doi: 10.1371/journal.pone.0236757 (PMC7392305; doi:10.1371/journal.pone.0236757)
Supplement: S2 Table — (DOCX) [file pone.0236757.s002.docx]

**S2 Table. Descriptive statistics for the model parameters**

| **Median(Std.Dev)** | **Long-term model** | **Short-term model** |
| --- | --- | --- |
| Intercept | -1.755(0.079) | -0.202(0.085) |
| Precision | 5.457(0.415) | 6.064(0.636) |
| Time | 0.062(0.002) | 0.066 (0.006) |
