# Supplementary material for: A Bayesian framework to unravel food, groundwater, and climate linkages: A case study from Louisiana
Source: PLoS One. 2020 Jul 30;15(7):e0236757. doi: 10.1371/journal.pone.0236757 (PMC7392305; doi:10.1371/journal.pone.0236757)
Supplement: S3 Table — (DOCX) [file pone.0236757.s003.docx]

**S3 Table** **Farm-related annual mean income for the study counties**

| Year | Annual Mean Income($) |
| --- | --- |
| 1997 | 1026600 |
| 2002 | 1316800 |
| 2007 | 1502000 |
| 2012 | 2263500 |
