# Supplementary material for: A Bayesian framework to unravel food, groundwater, and climate linkages: A case study from Louisiana
Source: PLoS One. 2020 Jul 30;15(7):e0236757. doi: 10.1371/journal.pone.0236757 (PMC7392305; doi:10.1371/journal.pone.0236757)
Supplement: S1 Fig — (DOCX) [file pone.0236757.s004.docx]

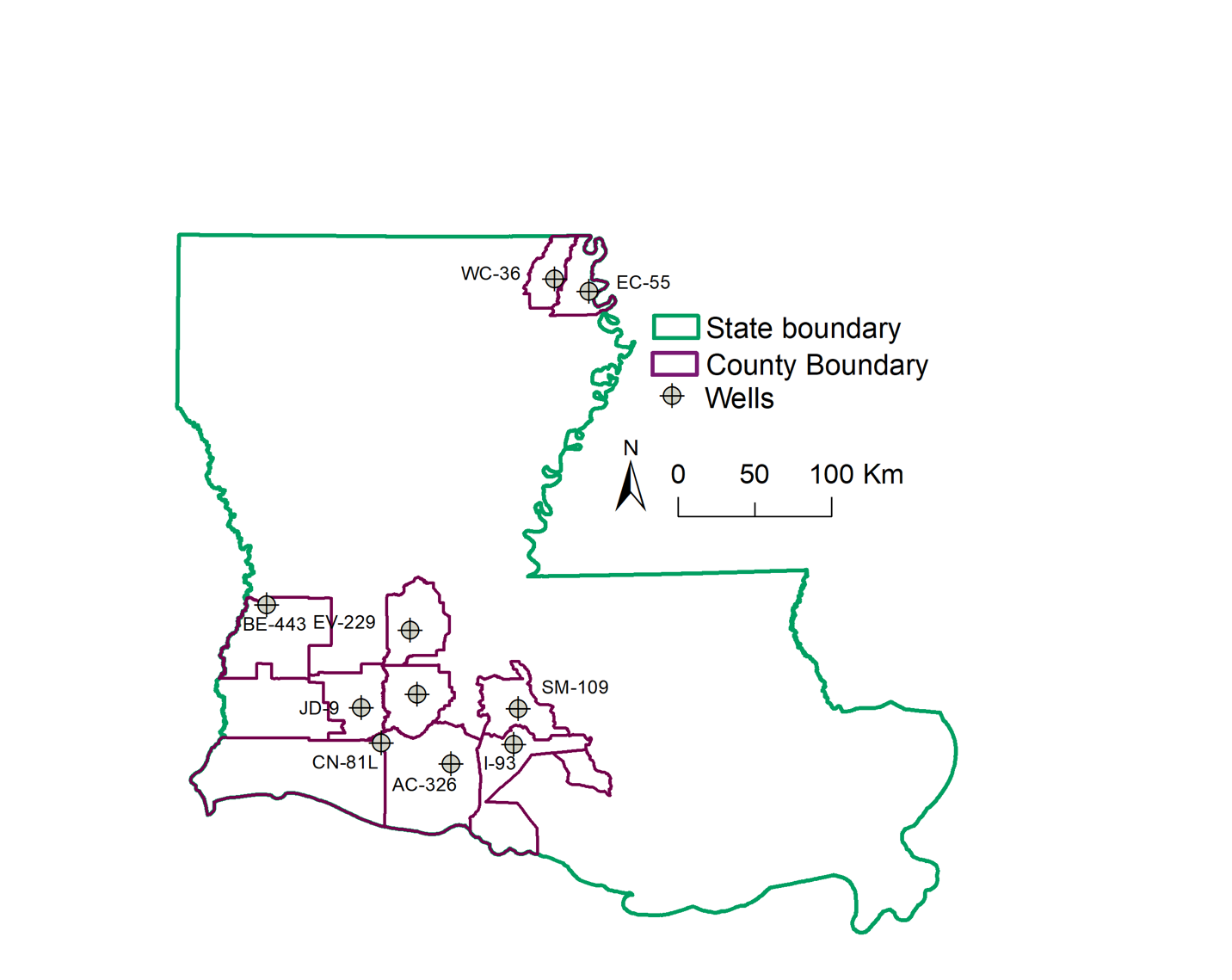


**S1 Fig.** **A map of location of study groundwater wells along with the county and state boundaries for Louisiana.**
