# Supplementary material for: A Bayesian framework to unravel food, groundwater, and climate linkages: A case study from Louisiana
Source: PLoS One. 2020 Jul 30;15(7):e0236757. doi: 10.1371/journal.pone.0236757 (PMC7392305; doi:10.1371/journal.pone.0236757)
Supplement: S2 Fig — (DOCX) [file pone.0236757.s005.docx]

**S2 Fig.** **Temporal patterns of mean air temperature and rainfall total during growing season for the climate stations located in the southern and northern part of the Louisiana.**
