# Supplementary material for: A Bayesian framework to unravel food, groundwater, and climate linkages: A case study from Louisiana
Source: PLoS One. 2020 Jul 30;15(7):e0236757. doi: 10.1371/journal.pone.0236757 (PMC7392305; doi:10.1371/journal.pone.0236757)
Supplement: S3 Fig — (DOCX) [file pone.0236757.s006.docx]

**S3 Fig**. **Temporal patterns of oil prices (USD) observed during the study period.**
