# Supplementary material for: A Bayesian framework to unravel food, groundwater, and climate linkages: A case study from Louisiana
Source: PLoS One. 2020 Jul 30;15(7):e0236757. doi: 10.1371/journal.pone.0236757 (PMC7392305; doi:10.1371/journal.pone.0236757)
Supplement: S4 Fig — Black filled circle and associated thick black line represent median and 50% confidence interval, respectively. Abbreviations: Irrigation wells (Iwells), Area planted (AP), Groundwater level (GW), Mean Air temperature (Tmean), and area normalized Nitrogen fertilizers (N_fert). (DOCX) [file pone.0236757.s007.docx]

**S4 Fig. The posterior distributions of regression coefficients for the covariates used in the limited duration hierarchical Bayesian model 2 . Black filled circle and associated thick black line represent median and 50% confidence interval, respectively. Abbreviations: Irrigation wells (Iwells), Area planted (AP), Groundwater level (GW), Mean Air temperature (Tmean), and area normalized Nitrogen fertilizers (N_fert).**
